# Decrease in early mortality for newly diagnosed multiple myeloma patients in the Netherlands: a population-based study

**DOI:** 10.1038/s41408-021-00571-8

**Published:** 2021-11-11

**Authors:** Mirian Brink, Kaz Groen, Pieter Sonneveld, Monique C. Minnema, Annemiek Broijl, Avinash G. Dinmohamed, Ellen van der Spek, Mark-David Levin, Paula F. Ypma, Esther de Waal, Eduardus F. M. Ward Posthuma, Sonja Zweegman, Niels W. C. J. van de Donk

**Affiliations:** 1 Department of Research and Development, Netherlands Comprehensive Cancer Organisation (IKNL), Utrecht, The Netherlands; 2 Department of Hematology, Cancer Center Amsterdam, Amsterdam UMC, Vrije Universiteit Amsterdam, Amsterdam, The Netherlands; 3 Department of Hematology, Erasmus MC Cancer Institute, Rotterdam, The Netherlands; 4 Department of Hematology, Cancer Center, UMC Utrecht, Utrecht, The Netherlands; 5 Department of Public Health, Erasmus University Medical Center, Rotterdam, The Netherlands; 6 Department of Internal Medicine, Rijnstate Hospital, Arnhem, The Netherlands; 7 Department of Internal Medicine, Albert Schweitzer Hospital, Dordrecht, The Netherlands; 8 Department of Hematology, Haga Teaching Hospital, The Hague, The Netherlands; 9 Department of Internal Medicine, Medical Center Leeuwarden, Leeuwarden, The Netherlands; 10 Department of Internal Medicine, Reinier de Graaf Group, Delft, The Netherlands

**Keywords:** Prognosis, Risk factors

## Abstract

Identification of risk factors for early mortality (EM) in multiple myeloma (MM) patients may contribute to different therapeutic approaches in patients at risk for EM. This population-based study aimed to assess trends in EM and risk factors for EM among MM patients diagnosed in the Netherlands. All MM patients, newly diagnosed between 1989 and 2018, were identified in the Netherlands Cancer Registry. Patients were categorized into three calendar periods (1989–1998, 1999–2008, 2009–2018) and into five age groups (≤65, 66–70, 71–75, 76–80, >80 years). EM was defined as death by any cause ≤180 days post-diagnosis. We included 28,328 MM patients (median age 70 years; 55% males). EM decreased from 22% for patients diagnosed in 1989–1998 to 13% for patients diagnosed in 2009–2018 (*P* < 0.01) and this decrease was observed among all age groups. Exact causes of death could not be elucidated. Besides patient’s age, we found that features related to a more aggressive disease presentation, and patient characteristics reflecting patients’ physical condition were predictive of EM. In summary, EM decreased from 1999 onwards. Nevertheless, EM remains high, especially for patients aged >70 years. Therefore, novel strategies should be explored to improve the outcome of patients at risk for EM.

## Introduction

The introduction of novel agents and autologous stem cell transplantation (SCT) improved the population-level survival of patients with newly diagnosed multiple myeloma (MM). Nonetheless, early mortality (EM) remains a major clinical issue, especially in older patients. Understanding the determinants of EM may translate into improved supportive care and individualized treatment strategies. Risk factors for EM that have previously been identified in clinical trials include features related to aggressive disease presentation or high-risk MM and patient-related factors, such as age and comorbid conditions [1–4]. However, these clinical trial populations differ substantially from the general MM population due to limited enrollment of frail elderly patients and preclusion of patients with comorbidities or poor performance status at diagnosis [5]. Also, single- and multi-center studies, often performed in large academic hospitals, probably underestimate the proportion of patients with EM, as many elderly and frail patients are infrequently referred to a specialized myeloma center [2]. To date, the most far-reaching population-level study reporting on EM within 180 days post-diagnosis in MM patients originates from the Surveillance Epidemiology and End Results (SEER) database, including >90,000 MM patients between 1975 and 2015 [6]. Here, EM increased with advancing age. However, information on disease-related features was lacking. Moreover, across studies, there is variability in the definition of EM, mainly using cut-off marks of 60 [1, 7] or 180 days [5, 6, 8]. Altogether, this results in a rather broad range of EM estimates among the different studies.

In this nationwide, population-based study, we aimed to complement and expand on previously reported single- and multi-center studies, as well as the analysis from the SEER database, on trends and risk factors of EM among patients with MM seen in routine clinical practice in the Netherlands. We included all patients diagnosed between 1989 and 2018 including patients who never initiated anti-MM therapy and are typically excluded in other studies.

## Methods

### Registry and study population

We identified all patients with newly diagnosed MM ≥18 years diagnosed between 1989 and 2018—with survival follow-up through February 1, 2021—from the nationwide Netherlands Cancer Registry (NCR), using ICD-O morphology code 9732, which ascertains all newly diagnosed malignancies in the Netherlands since 1989 through multiple notification sources. Information on dates of birth and diagnosis, sex, disease topography and morphology, hospital of diagnosis, and prior malignancies is routinely recorded in the NCR by trained registrars of the NCR through retrospective medical record review. Seventy-three patients diagnosed through autopsy were excluded from all analyses.

For MM patients diagnosed as of January 1, 2014, additional, more detailed information on World Health Organization (WHO) performance status, type of M-protein, M-protein level, bone marrow plasma cell percentage, platelet count, cytogenetic risk, and the number of bone lesions, as well as levels of serum albumin, serum β2-microglobulin, serum calcium, serum creatinine, and hemoglobin, was recorded in the NCR. Moreover, the type and number of novel agents and reason not to start therapy were available. For patients who never received first-line therapy, we identified the reason why a newly diagnosed patient did not receive this treatment. The type and number of novel agents (i.e., bortezomib, thalidomide, lenalidomide, and daratumumab) incorporated into a regimen are presented separately for patients with or without EM according to the five age groups.

The Supplemental Materials provides details about the registry, as well as additional information on baseline patient characteristics, disease features, and treatment-related aspects, as collected in the current study. In the remaining text, we will refer to study period 1989–2018 as cohort 1 and study period 2014–2018 as sub-cohort 1a.

According to the Central Committee on Research involving Human Subjects (CCMO), this type of observational study does not require approval from an ethics committee in the Netherlands. The Privacy Review Board of the NCR approved the use of anonymous data for this study.

### Statistical analyses

For cohort 1, MM patients were stratified according to year of diagnosis by using three calendar periods (i.e., 1989–1998, 1999–2008, and 2009–2018). Pearson chi-square test was used to compare categorical covariates, and Kruskal–Wallis test was used to compare non-normally distributed continuous covariates across the three calendar periods in cohort 1.

Considering that our main focus was to study the trends of EM over time as well as impact of patient- and tumor-related factors on EM and that it can take 2–4 months for treatment regimens to show steady-state benefit, we defined EM as all-cause death within 180 days post-diagnosis across 1989–2018. By using this cut-off mark, we were able to compare our results with those obtained in several larger studies, including the SEER population-based study that used a similar cut-off [5, 6, 8]. EM estimates were presented overall, as well as separately for the three calendar periods in cohort 1 and for the five age groups (i.e., 18–65, 66–70, 71–75, 76–80, >80 years) in cohort 1 and sub-cohort 1a.

For cohort 1, we evaluated the impact of age, sex, period of diagnosis, hospital type at diagnosis, and prior malignancies on EM by calculating hazard ratios (HRs) and corresponding 95% confidence intervals (95% CIs) using univariable and multivariable Cox proportional regression analysis. For sub-cohort 1a, we evaluated the impact of additional disease-related features at baseline, such as type of M-protein, bone marrow plasma cell percentage, thrombocytopenia, cytogenetic risk, and the number of bone lesions, as well as levels of serum albumin, serum β2-microglobulin, serum calcium, serum creatinine, and hemoglobin, using the same strategy as in cohort 1.

We calculated relative survival (RS) to estimate the disease-specific survival in the absence of information on the cause of death. As cause of death information is unavailable in the NCR, we were unable to compute disease-specific survival. Therefore, we employed RS since it estimates disease-specific survival but does not require cause of death information. RS is defined as the ratio of the overall survival (OS) of the patient cohort to the expected OS of an equivalent group from the general population, matched to the patients by age, sex, and calendar year. As such, RS reflects the overall excess mortality associated with an MM diagnosis. The expected OS was estimated as per the Ederer II methodology using Dutch population life tables, stratified by age, sex, and calendar year. RS rates with 95% CIs were calculated at 5 years post-diagnosis for the five age groups, stratified by three calendar periods, and measured from the time of diagnosis until death, emigration, or end of follow-up (February 1, 2021), whichever came first.

A *P* value <0.05 was considered statistically significant. All analyses were performed using STATA/SE 17.0 (StataCorp LP, College Station, TX, USA).

## Results

### Patient characteristics

Between 1989 and 2018, 28,328 MM patients were diagnosed in the Netherlands (cohort 1). Overall, the median age at diagnosis was 70 years (range, 25–99 years), 55% was male, 88% were diagnosed in non-academic centers, and 13% had prior malignancies. Baseline characteristics, according to the calendar period of diagnosis, are presented in Table 1. The age distribution and the proportion of patients diagnosed in non-academic centers remained similar over time.

**Table 1:** Baseline characteristics of patients diagnosed in 1989–2018 in the Netherlands, stratified by calendar period.

	Calendar period		
	1989–1998	1999–2008	2009–2018
	*n* = 7,312	*n* = 8,822	*n* = 12,194
	No. (%)	No. (%)	No. (%)
Sex, male	3796 (52)	4830 (55)	6967 (57)
Age at diagnosis			
18–65 years	2498 (34)	3204 (36)	4210 (35)
66–70 years	1158 (16)	1311 (15)	2024 (17)
70–75 years	1309 (18)	1506 (17)	1981 (16)
76–80 years	1164 (16)	1433 (16)	2026 (17)
>80 years	1183 (16)	1368 (16)	1953 (16)
Median, range	70.5 (28–99)	70 (28–99)	70 (25–97)
Prior malignant disease, yes	572 (8)	1068 (12)	2163 (18)
Hospital of diagnosis			
Non-academic center	6524 (89)	7605 (86)	10,858 (89)
Academic center	788 (11)	1217 (14)	1336 (11)
Follow-up, months			
Median, range	25.4 (0.03–367.1)	32.4 (0.03–253.0)	33.9 (0.03–132.8)

To evaluate patients and myeloma-related characteristics in more detail, additional data from 5360 MM patients diagnosed in 2014–2018 (sub-cohort 1a) was available (summarized in Supplemental Table 1).

### Trends in EM and survival

**Fig. 1:**
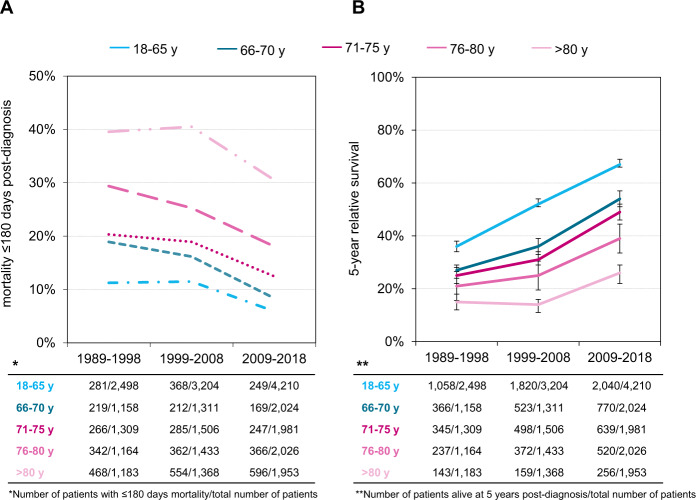
Trends in relative survival and early mortality of patients with multiple myeloma (MM) in the Netherlands, 1989–2018. **A** shows the trend in early mortality ≤180 days post-diagnosis according to age for the three consecutive calendar periods (1989–1998, 1999–2008, and 2009–2018). The absolute numbers of patient with early mortality ≤180 days post-diagnosis and the total number of patients per age group and calendar period are presented in the table below. **B** shows the 5-year relative survival estimates for MM patients diagnosed between January 1, 1989 and December 31, 2018 according to age. The absolute numbers of patients alive at 5 years post-diagnosis and the total number of patients per age group and calendar period are presented in the table below.

Of all the 28,328 patients in cohort 1, 4984 patients died ≤180 days post-diagnosis (18% of the entire patient’ population and 23% of all deaths). EM was lower in younger patients and decreased over time in all age categories. For patients aged 18–65 years, EM rates were 11, 11, and 6% for the three consecutive calendar periods (*P* < 0.01). The corresponding proportions for patients aged 66–70 years were 19, 16, and 8% (*P* < 0.01); for patients aged 71–75 years, 20, 19, and 12% (*P* < 0.01); for patients aged 76–80 years, 29, 25, and 18%; and for patients aged >80 years, 40, 41, and 31% (*P* < 0.01) (Fig. 1A). RS improved significantly across the three calendar periods, i.e., from 27% in 1989–1998 to 37% in 1999–2008 and 52% in 2009–2018. Stratified by the five age groups, the increase of RS from 1989–1998 to 2009–2018 was most pronounced for the younger patients, i.e., from 36 to 67% (31% increase) for patients aged 18–65 years, from 27 to 54% (27% increase) for patients aged 66–70 years, from 25 to 49% (24% increase) for patients 71–75 years, from 21 to 39% (18% increase) for patients 76–80 years, and from 15 to 26% (11% increase) for patients aged >80 years (Fig. 1B).

### Association between patient- and tumor-related risk factors with EM

In cohort 1, calendar period was an independent predictor of EM with a 50% lower risk of EM for patients diagnosed in the most recent calendar period 2009–2018, as compared to 1989–1998 (Supplemental Table 2). We also observed that increasing age and male sex were independently associated with increased EM risk.

To further explore the influence of baseline patient- and MM-related factors on EM, we used detailed data on patient and diagnostic parameters of 5360 NDMM patients in sub-cohort 1a (see Supplemental Table 1 for baseline characteristics). In this population, 753 patients (14%) died ≤180 days post-diagnosis. Patients with EM were older at diagnosis with EM rates of 5, 10, 14, 20, and 33% for the five consecutive age groups (*P* < 0.01). Univariable and multivariable HRs and the corresponding 95% CIs are shown in Table 2. In detail, compared to patients aged 18–65 years, multivariable analysis showed that risk of EM was 1.7 times higher for patients aged 66–70 years (HR, 1.72; 95% CI, 1.29–2.30), 2.6 times higher for patients aged 71–75 years (HR, 2.56; 95% CI, 1.95–3.36), 3.4 times higher for patients aged 76–80 years (HR, 3.41; 95% CI, 2.63–4.41), and 5.0 times higher for patients aged >80 years (HR, 5.03; 95% CI, 3.93–6.43). Apart from age, risk of EM was also independently associated with WHO performance score 3–4 (HR, 3.68; 95% CI, 2.71–4.99), ≥60% bone marrow plasma cells (HR, 1.19; 95% CI, 1.00–1.41), thrombocytopenia (HR, 1.76; 95% CI, 1.37–2.26), serum β2-microglobulin ≥3.5 mg/L (HR, 1.59; 95% CI, 1.21–2.10), albumin <35 g/L (HR, 1.72; 95% CI, 1.45–2.02), hypercalcemia (HR, 1.69; 95% CI, 1.44–1.99), renal impairment (HR, 1.42; 95% CI, 1.21–1.67), anemia (HR, 1.17; 95% CI, 1.00–1.36), and high-risk cytogenetics (HR, 1.25; 95% CI, 1.00–1.57) (Table 2, multivariable model).

**Table 2:** Impact of various diagnostic parameters on risk of EM for patients with newly diagnosed MM diagnosed in 2014–2018.

	Univariable		Multivariable	
	HR (95% CI)	*P*^g^	HR (95% CI)	*P*^g^
Age at diagnosis, years				
18–65	1 (reference)		1 (reference)	
66–70	1.89 (1.42–2.52)	**<0.01**	1.72 (1.29–2.30)	<**0.01**
71–75	2.78 (2.12–3.63)	**<0.01**	2.56 (1.95–3.36)	<**0.01**
76–80	4.15 (3.23–5.34)	**<0.01**	3.41 (2.63–4.41)	<**0.01**
>80	7.64 (6.06–9.63)	**<0.01**	5.03 (3.93–6.43)	**<0.01**
Gender				
Female	1 (reference)		—	—
Male	0.94 (0.81–1.08)	0.38	—	—
Hospital type of diagnosis				
Academic	1 (reference)		—	—
Non-academic	1.51 (1.14–2.01)	**<0.01**	—	—
Prior malignancy				
No	1 (reference)		—	—
Yes	1.45 (1.23–1.71)	**<0.01**	—	—
WHO performance score				
0–2	1 (reference)		1 (reference)	
3–4	6.06 (4.49–8.20)	**<0.01**	3.68 (2.71–4.99)	<**0.01**
Unknown	2.79 (2.34–3.32)	**<0.01**	1.95 (1.63–2.33)	**<0.01**
% plasma cells				
<60%	1 (reference)		1 (reference)	
≥60%	1.25 (1.05–1.47)	**0.01**	1.19 (1.00–1.41)	**0.05**
Unknown	4.00 (3.34–4.81)	**<0.01**	2.05 (1.68–2.50)	**<0.01**
Thrombocytopenia^a^				
No	1 (reference)		1 (reference)	
Yes	2.36 (1.85–3.00)	**<0.01**	1.76 (1.37–2.26)	**<0.01**
Unknown	0.74 (0.24–2.30)	0.60	0.49 (0.15–1.55)	0.22
Type of M-protein				
IgG	1 (reference)		—	—
IgA	1.00 (0.82–1.21)	0.99	—	—
LCD	1.00 (0.82–1.21)	0.97	—	—
Other	1.28 (0.94–1.74)	0.12	—	—
Unknown	3.30 (2.29–4.75)	**<0.01**	—	—
β2-microglobulin^b^				
<3.5 mg/L	1 (reference)		1 (reference)	
≥3.5 mg/L	3.33 (2.56–4.33)	**<0.01**	1.59 (1.21–2.10)	**<0.01**
Unknown	5.30 (4.05–6.93)	**<0.01**	2.05 (1.54–2.73)	**<0.01**
Albumin^b^				
≥35 g/L	1 (reference)		1 (reference)	
<35 g/L	2.71 (2.32–3.16	**<0.01**	1.72 (1.45–2.02)	**<0.01**
Unknown	1.15 (0.70–1.88)	0.58	0.76 (0.45–1.27)	0.30
Hypercalcemia^c^				
No	1 (reference)		1 (reference)	
Yes	2.36 (2.02–2.75)	**<0.01**	1.69 (1.44–1.99)	**<0.01**
Unknown	0.59 (0.30–1.20)	0.15	0.59 (0.29–1.21)	0.15
Poor renal function^d^				
No	1 (reference)		1 (reference)	
Yes	1.99 (1.70–2.32)	<**0.01**	1.42 (1.21–1.67)	**<0.01**
Unknown	0.37 (0.09–1.46)	0.16	0.59 (0.14–2.44)	0.47
Anemia^e^				
No	1 (reference)		1 (reference)	—
Yes	1.96 (1.70–2.27)	**<0.01**	1.17 (1.00–1.36)	**0.05**
Unknown	—	—	—	—
Bone lesion				
0	1 (reference)		—	—
≥1	0.86 (0.72–1.02)	0.09		
Unknown	3.23 (2.51–4.15)	**<0.01**		
Cytogenetic risk^f^				
Standard risk	1 (reference)		1 (reference)	
High risk	1.24 (0.99–1.55)	0.07	1.25 (1.00–1.57)	**0.05**
Unknown	2.87 (2.43–3.39)	**<0.01**	1.59 (1.33–1.91)	**<0.01**

*MM* multiple myeloma, *HR* hazard ratio, *CI* confidence interval, *LCD* light chain disease.

^a^Thrombocytopenia: thrombocytes <100 × 10^9^/L.

^b^The impact of the separate parameters of International Staging System (ISS) was evaluated rather than the ISS score.

^c^Hypercalcemia: serum calcium >2.75 mmol/L.

^d^Poor renal function: creatinine >177 mmol/L.

^e^Anemia: hemoglobin <6.2 mmol/L.

^f^High risk; presence of t(4;14), t(14;16), and/or del(17p), standard risk; presence of any other chromosomal aberration or without any aberration, unknown; no cytogenetic assessment performed.

^g^Statistically significant *P* values (*P* < 0.05) are presented in bold.

### First-line treatment and EM

**Fig. 2:**
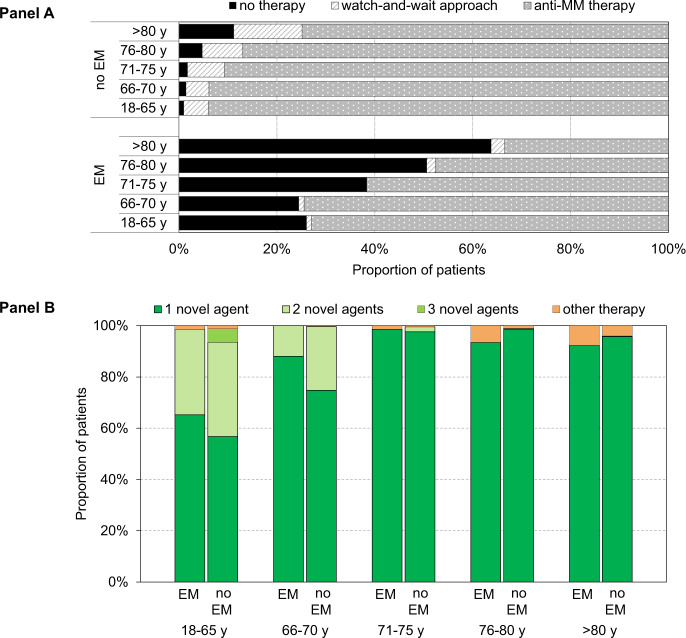
First-line treatment of patients with multiple myeloma (MM) in the Netherlands, 2014–2018. **A** shows the proportions of MM patients who never received first-line treatment, who were offered a watch-and-wait-approach, or who received anti-MM treatment, according to occurrence of EM and age at diagnosis. **B** shows the type of first-line regimen (regimen containing 1, 2, 3, or no novel agents) for newly diagnosed MM patients who started anti-MM treatment, according to occurrence of EM and age at diagnosis.

To investigate the proportion of patients with EM for whom anti-myeloma therapy was never initiated and to assess the impact of type of therapy among newly diagnosed MM patients with or without EM, we used information on the type of the first-line regimen that was administered to the 5360 patients identified in sub-cohort 1a. Overall, 495 (9%) patients never received anti-MM therapy, of whom 357 patients (72%) died ≤180 days post-diagnosis. In addition, 4530 patients (85%) were initiated anti-MM therapy, and 335 patients (6%) were offered a wait-and-watch approach until disease progression or development of symptoms. For patients with a wait-and-watch approach, there was a lower tumor burden as compared to patients who never received therapy, as reflected by a lower proportion of patients with ≥60% bone marrow plasma cells (8 vs. 19%, respectively (*P* < 0.01)) and higher proportion of patients with International Staging System stage 1 (19 vs. 6%, respectively (*P* < 0.01)). In Fig. 2A, the proportions of patients who never received first-line treatment, who were offered a watch-and-wait approach, or who received anti-MM therapy are presented according to age at diagnosis and stratified for the occurrence of EM. As expected, the proportion of patients who never received therapy increased with older age. Also, for each age category, the proportion of MM patients never receiving therapy was significantly higher in patients who experienced EM than patients without EM (*P* = 0.05). The main reasons for not starting first-line therapy in patients who experienced EM were poor functional status at diagnosis (36%), explicit wish of the patient (27%), poor prognosis (14%), comorbidity (9%), other reasons (13%), and unknown (1%). Moreover, the proportion of patients who were offered a watch-and-wait approach increased with older age, but only for patients without EM (Fig. 2A).

Finally, we restricted our analysis to patients who started first-line treatment. The proportion of patients treated with a regimen containing 1, 2, 3, or no novel agents (other therapy) is depicted in Fig. 2B, and these proportions are presented according to the five age groups and occurrence of EM. Across all age groups, most patients received a regimen containing one novel agent. With increasing age, the proportion of patients who received a regimen containing 2 or 3 novel agents decreased from 42% for patients aged 18–65 years to 0% for patients aged >80 years. In fact, there was a modestly more frequent use of regimens without novel agents in patients aged >80 years as compared to patients aged 18–65 years, i.e., 5 vs. 1% (*P* < 0.01). The proportion of patients aged 18–65 years who received a regimen containing 1 or 2 novel agents was similar between patients with and without EM (*P* = 0.26). However, 5% of the patients without EM in this age group received a regimen with 3 novel agents, as compared to none of the patients who experienced EM. There was no difference in number of novel agents used as part of first-line therapy between patients with or without EM aged 66–70 years, 71–75 years, or >80 years. However, the use of regimens without novel agents was significantly higher in patients aged 76–80 years, who experienced EM, as compared to patients without EM (6 vs. 1%; *P* < 0.01). We did not observe differences in the use of bortezomib-, lenalidomide-, daratumumab-, or thalidomide-containing regimens in first-line treatment among patients with or without occurrence of EM, neither when age categories were analyzed separately.

## Discussion

In this nationwide, population-based study among patients diagnosed with MM during a 30-year period in the Netherlands, we demonstrate that survival of MM patients improved over time and that the EM rate markedly decreased across all age groups. Nevertheless, EM remains high for older patients, especially patients aged >70 years, probably because of a higher likelihood of disease- and/or treatment-related complications in this more vulnerable patient population. This study is, to the best of our knowledge, the most extensive population-based study to date that offers comprehensive information on the frequency of and risk factors for EM, as well as the impact of first-line therapy on EM.

We set the cut-off for EM at 180 days post-diagnosis of MM to compare our results with those obtained in previous studies, which used a similar cut-off [3, 5, 6, 8]. Across these studies, there is considerable heterogeneity in proportions of EM. In a prospective registry with 1493 symptomatic MM patients diagnosed between 2009 and 2011 [5] and a retrospective study with 542 MM patients diagnosed between 2002 and 2014 [3], EM estimates were approximately 50% lower, compared to EM in MM patients diagnosed between 2009 and 2018 in our study. On the other hand, the EM estimates observed in a relatively small single center study with 122 MM patients diagnosed between 2007 and 2013 [8] and in the SEER database with 90,975 MM patients registered between 1975 and 2015 [6] were two times higher, when compared to our results. Explanations for the rather broad range of EM estimates may lie in differences between the evaluated patient populations, including the number of patients analyzed in the different studies, differences in treatment and supportive care [3, 5, 6, 8], and, in some studies, exclusion of frail patients, such as elderly patients or patients who never received anti-MM treatment. Our study is one of the first population-based studies that includes all MM patients, i.e., irrespective of age and/or eligibility to receive induction therapy, and therefore our analysis represents a real-world population with an overall EM of 13% in patients diagnosed in the most recent calendar period 2009–2018.

We demonstrate significant improvements in survival over the past 30 years among MM patients across all different age categories, with major improvements since 2009. These impressive improvements are most likely related to the administration of novel agents, such as immunomodulatory drugs and proteasome inhibitors, and most recently monoclonal antibodies, as well as introduction of autologous SCT and supportive treatment measures. The extent of this improvement, however, is more notable in younger patients than in older patients, likely due to lower tolerance to multidrug regimens in elderly patients. Also, EM markedly decreased since 2009, which can be explained by the superior activity and better tolerability of newer regimens, as well as improvements in supportive care. In addition, an increased use of dose-adapted regimens in intermediate-fit and frail patients may also have contributed to reduced EM over time [9–11]. The importance of dose on clinical outcomes has been clearly demonstrated for dexamethasone, with a randomized phase 3 study showing that lenalidomide plus low-dose dexamethasone resulted in lower toxicity, including infections and venous thromboembolic events, which translated into improved short-term survival, compared to lenalidomide plus high-dose dexamethasone in patients with newly diagnosed MM [11]. However, the proportion of elderly patients, who died early after diagnosis, remains significantly higher than what is observed in younger patients. Apart from age, other determinants of EM identified in our study include features related to a more aggressive disease presentation (e.g., hypercalcemia, renal impairment, and anemia), high disease burden (e.g., ≥60% bone marrow plasma cells, thrombocytopenia, low albumin, and elevated β2-microglobulin), high-risk cytogenetics, and characteristics reflecting the patients’ physical condition (e.g., WHO performance status 3–4). Our findings are in line with several other studies [1, 5, 8] showing that the risk of EM is dependent on patient characteristics, including age as well as tumor-related features, such as albumin and β2-microglobulin levels. Altogether, better identification of patients at high risk of EM may result in improved and more tailored management of newly diagnosed MM patients [7]. Because infections and cardiovascular disease are major causes of EM [7, 12], patients at high risk of EM should receive adequate supportive care, including antibacterial and thrombosis prophylaxis [13]. In addition, older and less fit patients may benefit from treatment strategies adjusted by dose and schedule in order to improve tolerability and prevent treatment discontinuation [14]. These new treatment strategies should preferentially be evaluated in the context of clinical trials.

Because in our analysis EM is more common in elderly patients and in those with aggressive presentation and with characteristics reflecting frailty, it is not unexpected that a substantial proportion of patients with EM never started anti-MM therapy. Prevention of diagnostic delay leading to earlier diagnosis and development of new regimens, which are both highly active and well tolerated, may further improve outcomes in these patients. We also observed two relatively modest differences in type of first-line therapy in patients, with or without EM, who did initiate first-line treatment. First, there was a modestly higher use of regimens containing 3 different novel agents among patients aged 18–65 years without EM. Second, the use of regimens without novel agents was more common in patients aged ≥76 years who experienced EM. These differences may reflect the differential ability of regimens to rapidly and safely control disease, but there may also be a bias toward the use of more effective regimens, resulting in rapid disease control in fit patients. Cause of death in patients with EM who received therapy is probably related to the presence of refractory disease and/or treatment-related complications, but data on this important topic was not available in the registry.

The main strength of this study is the use of a nationwide population-based cancer registry over patients enrolled in clinical trials, which are characterized by substantial overrepresentation of fit patients. Limitations of our study mainly pertain to the lack of detailed information on tumor and patient characteristics throughout most of the study period (i.e., 1989–2013). In addition, we cannot exclude that some patients with smoldering MM (SMM), defined according to the most recent International Myeloma Working Group definition [15], were included in cohort 1. However, by using additional, more detailed information, which was collected in the NCR as of year of diagnosis 2014, we were able to exclude SMM patients in sub-cohort 1a. Moreover, information on exact cause of death was lacking in the NCR. Other studies have shown that infections, cardiovascular disease, and renal failure are major causes of early death in newly diagnosed MM patients [1, 7]. Identification of predictive factors for specific causes of early death may lead to better, individualized therapeutic interventions to avoid early toxicities during treatment, particularly in elderly patients. Despite these limitations, this cancer registry represents an important tool to gain insight into the outcome of large numbers of unselected patients—including subgroups of patients who are usually underrepresented due to frailty, advanced age, and/or comorbidities—since all newly diagnosed patients are captured, including those who never received anti-MM treatment.

In summary, population-level EM among MM patients decreased from 1999 onwards. Notwithstanding this encouraging finding, EM remains high in patients aged >70 years. Therefore, the design and conduct of forthcoming prospective intervention studies for patients at risk for EM are essential to establish recommendations for better supportive care or individualized, less toxic, anti-MM therapies to further reduce EM in this patient population. Collectively, MM patients at risk for EM may likely benefit from more rigorous supportive care measures or frailty-adapted therapeutic approaches.

## Supplementary information

Supplemental
