# Supplementary material for: Decrease in early mortality for newly diagnosed multiple myeloma patients in the Netherlands: a population-based study
Source: Blood Cancer J. 2021 Nov 11;11(11):178. doi: 10.1038/s41408-021-00571-8 (PMC8586330; doi:10.1038/s41408-021-00571-8)
Supplement: Supplementary file 1 — Supplemental [file 41408_2021_571_MOESM1_ESM.docx]

**SUPPLEMENTAL METHODS**

*The Netherlands Cancer Registry*

Nationwide since 1989, the population-based Netherlands Cancer Registry (NCR), which is maintained and hosted by the Netherlands Comprehensive Cancer Organisation (IKNL), has a coverage of at least 95% of all malignancies in the Netherlands.^1^ All newly diagnosed malignancies in the Netherlands are reported to the NCR via the Nationwide Network of Histopathology and Cytopathology, and the National Registry of Hospital Discharges (i.e. inpatient and outpatient discharges). Information on dates of birth and diagnosis, sex, disease topography and morphology, hospital of diagnosis, and prior malignancies is routinely recorded in the NCR by trained registrars of the NCR through retrospective medical records review. Topography and morphology are coded according to the International Classification of Diseases for Oncology (ICD-O). Information on the last known vital status for all patients (i.e. alive, death, or emigration) is obtained through annual linkage with the Nationwide Population Registries Network that holds vital statistics on all residents in the Netherlands. Prior malignancies were classified as malignancies with a date of diagnosis prior to MM date of diagnosis according to the third edition of the ICD-O. Basal cell carcinomas were not recorded in the NCR.

For all hematological malignancies diagnosed as of January 1, 2014, additional, more detailed, information on baseline patient characteristics, disease features, and treatment is routinely recorded in the NCR. For MM, this includes World Health Organisation (WHO) performance status, type of M-protein, M-protein level, bone marrow plasma cell percentage, platelet count, cytogenetic risk, number of bone lesions, as well as levels of serum albumin, serum β2-microglobulin, serum calcium, serum creatinine, and hemoglobin. Also information on first-line therapy (such as type and number of novel agents) or reason not to start therapy was available.

*Risk factors*

Thrombocytopenia was defined as platelet count <100*10^9^/L, hypercalcemia as serum calcium levels >2.75 mmol/L, poor renal function as creatinine level >177 mmol/L and anemia as hemoglobin level <6.2 mmol/L. Cytogenetic risk was classified as standard or high risk based on conventional cytogenetic studies or fluorescent in situ hybridization performed on purified plasma cells. Patients with a t(4;14), t(14;16) and/or del(17p) were classified as high-risk. Normal cytogenetics and other cytogenetic abnormalities were classified as standard risk. ISS stage 1 was defined as serum β2-microglobuline <3.50 mg/L and serum albumine≥35 g/L, ISS stage 3 as serum β2-microglobuline ≥5.50 mg/L, regardless serum albumin levels, and ISS stage 2 was defined as neither stage 1 nor 3. ISS stage unknown was defined as serum β2-microglobuline and/or serum albumin unknown.

*Exact therapeutic regimen*

Treatment with bortezomib, lenalidomide, daratumumab and/or thalidomide within 6 months post-diagnosis was defined as treatment with at least one cycle of a bortezomib-, lenalidomide-, daratumumab- or thalidomide-containing regimen, respectively. In the period 2014-2018, upfront treatment with a daratumumab-based regimen was rare (1-2%), because daratumumab-containing regimens for newly diagnosed patients were not yet reimbursed. We also differentiated between regimens containing 1, 2, 3 novel agents or other agents (0 novel agents).

**Supplemental references**

1. Schouten LJ, Hoppener P, van den Brandt PA, Knottnerus JA, Jager JJ. Completeness of cancer registration in Limburg, The Netherlands. *Int J Epidemiol* 1993;22(3):369-376.

**Supplemental results**

**Supplemental Table 1.** Baseline characteristics of MM patients diagnosed in

2014-2018 in the Netherlands, according to EM.

|  | **Patients with EM** | | | | **Patients without EM** | |
| --- | --- | --- | --- | --- | --- | --- |
|  | **No.** | **(%)** | | | **No.** | **(%)** |
| **Total No. of patients** | 754 |  | | | 4,606 |  |
| **Sex, male** | 424 | (56) | | | 2,677 | (58) |
| **Age, years** |  |  | | |  |  |
| 18-65 | 96 | (13) | | | 1,756 | (38) |
| 66-70 | 90 | (12) | | | 851 | (19) |
| 71-75 | 120 | (16) | | | 751 | (16) |
| 76-80 | 164 | (22) | | | 675 | (15) |
| >80 | 284 | (38) | | | 573 | (12) |
| Median, range | 78 (41-96) | | | | 69 (25-96) | |
| **Hospital of diagnosis** |  | |  |  |  |  |
| Non-academic center | 702 | (93) | | | 4,134 | (90) |
| Academic center | 52 | (7) | | | 472 | (10) |
| **Prior malignant disease, yes** | 189 | (25) | | | 845 | (18) |
| **WHO Performance score** |  |  | | |  |  |
| 0-2 | 165 | (22) | | | 2,148 | (47) |
| 3-4 | 57 | (8) | | | 100 | (2) |
| Unknown | 532 | (70) | | | 2,358 | (51) |
| **% BM Plasma cells** |  |  | | |  |  |
| <60% | 364 | (48) | | | 2,911 | (63) |
| ≥60% | 222 | (29) | | | 1,404 | (31) |
| Unknown | 168 | (22) | | | 291 | (6) |
| **Type of M-protein** |  |  | | |  |  |
| IgG | 404 | (54) | | | 2,578 | (56) |
| IgA | 141 | (19) | | | 903 | (19) |
| LCD | 134 | (18) | | | 857 | (19) |
| Other | 44 | (6) | | | 215 | (5) |
| Unknown | 31 | (4) | | | 53 | (1) |
| **β2-microglobuline** |  |  | | |  |  |
| <3.5 mg/L | 65 | (8) | | | 1,320 | (29) |
| ≥3.5 mg/L | 390 | (52) | | | 2,238 | (48) |
| Unknown | 299 | (40) | | | 1,048 | (23) |
| **Albumin** |  |  | | |  |  |
| <35 g/L | 504 | (67) | | | 1,900 | (41) |
| ≥35 g/L | 233 | (31) | | | 2,544 | (55) |
| Unknown | 17 | (2) | | | 162 | (4) |
| **ISS^1^** |  |  | | |  |  |
| 1 | 29 | (4) | | | 914 | (20) |
| 2 | 138 | (18) | | | 1,365 | (29) |
| 3 | 287 | (38) | | | 1,226 | (27) |
| Unknown | 300 | (40) | | | 1,101 | (24) |
| **Hypercalcemia^2^, yes** | 239 | (32) | | | 707 | (15) |
| **Renal insufficiency^3^, yes** | 228 | (30) | | | 783 | (17) |
| **Anemia^4^, yes** | 371 | (49) | | | 1,467 | (32) |
| **Thrombocytopenia^5^, yes** | 73 | (10) | | | 179 | (4) |
| **≥1 Bone lesions, yes** | 486 | (64) | | | 3,423 | (74) |
| **Cytogenetic risk^6^** |  |  | | |  |  |
| Standard-risk | 201 | (27) | | | 2,115 | (46) |
| High-risk | 120 | (16) | | | 1,011 | (22) |
| Unknown | 433 | (57) | | | 1,480 | (32) |

Abbreviations: BM, bone marrow; IQR; inter quartile range, ISS; International Staging System, CRAB; Calcium Renal Anemia Bone laesions

^1^ISS stage 1: serum β2-microglobuline<3.50 mg/L & serum albumine≥35 g/L; ISS stage 3: serum β2-microglobuline≥5.50 mg/L; ISS stage 2; neither stage 1 nor 3; unknown: serum β2-microglobuline and/or serum albumin unknown.

^2^Serum calcium>2.75 mmol/L.

^3^Creatinine>177 mmol/L.

^4^Hemoglobin<6.2 mmol/L.

^5^Thrombocytes<100*10^9^/L.

^6^High-risk presence of translocation (4;14), translocation (14;16) and/or deletion 17p; standard-risk: all other aberrations or no abnormalities.

**Supplemental Table 2.** Impact of period of diagnosis, sex, hospital type at diagnosis, prior malignancies, and anti-myeloma treatment on risk of early mortality for patients with MM diagnosed in 1989-2018.

|  | **Univariable** | | **Multivariable** | |
| --- | --- | --- | --- | --- |
|  | **HR (95%CI)** | ***P*** | **HR (95%CI)** | ***P*** |
| **Period of diagnosis**  1989-1998  1999-2008  2009-2018 | 1 (reference)  0.93 (0.87-0.99)  0.59 (0.55-0.63) | **0.03**  **<0.01** | 1 (reference)  0.94 (0.88-1.00)  0.58 (0.54-0.62) | **0.06**  **<0.01** |
| **Age at diagnosis**  18-65 years  66-70 years  71-75 years  76-80 years  >80 years | 1 (reference)  1.51 (1.36-1.68)  1.92 (1.75-2.11)  2.80 (2.57-3.06)  4.70 (4.34-5.10) | **<0.01**  **<0.01**  **<0.01**  **<0.01** | 1 (reference)  1.55 (1.39-1.71)  1.93 (1.75-2.12)  2.88 (2.63-3.14)  4.87 (4.48-5.28) | **<0.01**  **<0.01**  **<0.01**  **<0.01** |
| **Sex**  **Female**  Male | 1 (reference)  1.00 (0.95-1.06) | 0.94 | 1 (reference)  1.17 (1.11-1.24) | **<0.01** |
| **Hospital type at diagnosis**  Academic  Non-academic | 1 (reference)  1.41 (1.28-1.55) | **<0.01** | - | - |
| **Prior malignancy**  No  Yes | 1 (reference)  1.27 (1.17-1.37) | **<0.01** | - | - |

Abbreviations: HR; hazard ratio, CI; confidence interval
